# Stromal, rather than epithelial cyclooxygenase-2 (COX-2) expression is associated with overall survival of breast cancer patients

**DOI:** 10.1186/1471-2407-14-732

**Published:** 2014-09-30

**Authors:** Justyna Urban, Łukasz Kuźbicki, Grzegorz Szatkowski, Agata Stanek-Widera, Dariusz Lange, Barbara W Chwirot

**Affiliations:** Department of Medical Biology, Faculty of Biology and Environment Protection, Nicolaus Copernicus University, Lwowska 1, 87-100 Toruń, Poland; Department of Tumor Pathology, Maria Skłodowska-Curie Memorial Cancer Center and Institute of Oncology, Wybrzeże Armii Krajowej 15, 44-101 Gliwice, Poland

**Keywords:** Cyclooxygenase-2 (COX-2), Breast cancer, Tumour stroma, Patient survival

## Abstract

**Background:**

Prognostic value of enhanced COX-2 expression in breast cancer has been controversial for a long time. The opinions vary widely between studies. Moreover, significant majority of studies considered only COX-2 expression in cancer epithelial cells.

**Methods:**

We examined the prognostic value of COX-2 expression in both epithelial and stromal cells using three different antibodies and three algorithms of immunohistochemical scoring and categorizing the tumours into COX-2 overexpressing groups.

**Results:**

Our results demonstrate that COX-2 expression in stromal cells is independent prognostic factor indicating worse overall survival of patients. Such a result was obtained using each of the three antibodies and two of the algorithms used for evaluations of COX-2 expression levels. We also show that immunohistochemical assessment of the prognostic value of COX-2 expression in cancer epithelial cells depends to a large extent on a combination of primary antibodies and algorithms used for determination of the COX-2 over-expressing tumours.

**Conclusions:**

Our results indicate that stromal expression of COX-2 is independent prognostic parameter relatively insensitive to variations in sensitivity of antibodies used for its determination. Wide scatter of the published results concerning prognostic value of COX-2 expression in breast cancer tissues seems to be due to a large extent to multitude of antibodies and scoring algorithms used by different groups.

## Background

Increased expression of cyclooxygenase-2 (COX-2) has been reported for many types of human cancer including breast cancer. Moreover, several epidemiologic studies indicate that regular use of non-steroidal anti-inflammatory drugs (NSAIDs) which inhibit COX-2 reduces incidence of at least some types of human cancers like sporadic and familial colon cancer, pancreatic cancer, melanoma and breast cancer (see for instance [1, 2]). Large cohort study involving ca. 80 000 postmenopausal women showed 21%-28% reduction in the risk of breast cancer for women taking NSAIDs at least twice a week for 5–10 years [1].

A role of enhanced COX-2 expression in breast cancer development and progression has not been fully elucidated yet and the literature data on prognostic usefulness of COX-2 for the breast cancer are inconsistent. Several studies associated enhanced COX-2 expression with a worse survival of patients [3–16]. Other groups, however, reported that immunohistochemically detected COX-2 expression did not provide prognostic information [17–21].

COX-2 expression in mammary epithelial cells can be induced in several ways, among others by estrogen receptors (ER) [22]. On the other hand, COX-2 catalyzes production of prostaglandins which stimulate aromatase converting androgens to estrogens [23]. Park et al. [21] investigated prognostic value of COX-2 for cancers with and without ER expression and did not find evidence of decreased survival while similar studies of other authors lead to a conclusion that increased COX-2 expression is associated with a worse survival of patients with ER-negative breast cancers [7, 15]. According to immunohistochemical study of Chuah et al. [11] of tumours of patients subject to neoadjuvant chemotherapy a low COX-2 expression was associated with a better survival but only within patients with ER positive tumours.

Although some studies indicated prognostic value of COX-2 expression for the breast cancer using univariate statistical analyses such conclusions were not confirmed by multivariate analyses including several significant variables [3, 12–14]. Only Denkert et al. [4] found a correlation between COX-2 expression and survival in multivariate analysis. It should also be noticed that majority of the authors focused on the COX-2 expression in epithelial cells of the lesions and did not examine a role of stromal components. Other studies demonstrated, however, that outcomes of breast cancer patients might be assessed through examination of stromal biomarkers [24] and the study of Richardsen et al. [16] showed that indeed COX-2 expression in stroma but not in the epithelial cells was correlated with a survival of breast cancer patients.

Finally, a validity of immunohistochemical analyses depends to a large extent upon antibody specificity (see for instance [25–27]). Other factors important for a comparability of the data reported by different groups are sensitivity of antigen detection and differences in algorithms used for immunohistochemical scoring. In this work we used three different primary antibodies and three different immunohistochemical scoring systems to assess the expression of the COX-2 protein both in the cancer epithelial cells and in the stroma within the same set of breast cancer samples.

## Methods

### Patient material

The material for study was formalin fixed, paraffin embedded tissue samples obtained from 41 breast cancer patients who underwent surgery without neoadjuvant chemotherapy. The study consisted of 41 primary breast tumours of different expression of steroid hormones receptors (estrogen - ER, progesterone - PR), HER-2, size (pT1 – 16, pT2 – 14, pT3 – 1, pT4 – 6), and presence of metastases (N0 – 27, N1 – 9, N2 – 3). Tissue specimens were obtained from the archives of The Tumour Pathology Department, Maria Skłodowska-Curie Memorial Institute in Gliwice, Poland. The research has been approved by the Bioethics Committee at Oncology Center (Ref. No KB/430-27/14), Gliwice Division, Gliwice, Poland.

### Antibodies

COX-2 expression was detected using paralelly three types of primary antibodies: (i) Ab1 - rabbit monoclonal antibody binding to peptide mapped at the C-end of the COX-2 protein (SP21 clone: RM-1921, Thermo Fisher Scientific, Waltham, Massachusetts, USA). The same type of the antibody was used by the groups reporting contradictory results [12, 15]; (ii) Ab2 - mouse monoclonal antibody detecting peptide mapped at the C-end of the COX-2 protein (580–599 aa) (CX229 clone, 160112, Cayman Chemical, Ann Arbor, Michigan, USA). This type of the antibody has been most commonly used in the studies of COX-2 expression in breast lesions (at least 30 different studies, among them [3]); (iii) Ab3 - goat polyclonal antibody generated against peptide mapped at the N-end of the COX-2 protein (N-20, sc-1746, Santa Cruz Biotechnology, Santa Cruz, California, USA) used in the earlier studies of our group. Primary goat polyclonal anti-Cox-2 antibody from Santa Cruz Biotechnology was also applied in the study carried out by Richardsen et al. [16].

### Immunohistochemistry

The 3 μm sections were dewaxed and rehydrated in usual manner. The reagents and conditions of the immunohistochemical procedures are given in Table 1. We would like to notice that the typically brown product of DAB reaction acquires a blue to black color in the presence of nickel ions. Negative control reactions were carried out with PBS pH 7.4 devoid of primary antibodies. Appropriate mixtures of the antibodies and of the corresponding blocking peptides (Ab2 – Cayman Chemical, 360107; Ab3 – Santa Cruz Biotechnology, (N20) P, sc-1746 P), prepared according to the manufacturer’s instruction, were used to evaluate the specificity of the antibodies in immunohistochemical assays.

**Table 1:** **Reagents and conditions of the immunohistochemical detection of COX-2 in formalin-fixed paraffin-embedded breast cancer lesions**

	Ab1	Ab2	Ab3
Antigen retrieval	heating up in 0.01 M citrate buffer pH 6.0 in a microwave oven (650 W) for 10 minutes		
Blocking serum*	1.5% normal goat serum	1.5% normal horse serum	1.5% normal rabbit serum
Type of primary antibody	rabbit monoclonal antibody (SP21 clone, RM-1921, Thermo Fisher Scientific, Waltham, Massachusetts, USA)	mouse monoclonal antibody (CX229 clone, 160112, Cayman Chemical, Ann Arbor, Michigan, USA)	goat polyclonal antibody (N-20, sc-1746, Santa Cruz Biotechnology, Santa Cruz, California, USA)
Antibody dilution (v/v in PBS pH 7.4)	1:100	1:200	1:500
Incubation period	overnight at 4°C, in a humid chamber		
Antibody detection system*	Vectastain Elite ABC Kits appropriate for each type of primary antibodies		
Peroxidase substrate	0.05% 3,3′ diaminobenzidine (DAB, Sigma-Aldrich, St. Louis, Missouri, USA) with 0.01% H_2_O_2_ and 0.06% NiCl_2_ (Sigma-Aldrich) in Tris–HCl buffer, pH 7.4		
Counterstaining*	nuclear red		

*(Vector Laboratories, Burlingame, California, USA).

Negative control sections did not produce detectable DAB precipitates. The expression of COX-2 by keratinocytes of normal human skin was considered positive control.

Microscopic examinations and digital imaging were carried out using the BX41 microscope with white-light illumination (Olympus Optical Co., Tokyo, Japan) equipped with digital camera and image analysis software (analySIS 3.2, Soft Imaging System, Hanover, Germany).

### Evaluation of immunostaining

Immunohistochemical scoring was carried out for ten fields of view at 400× magnification and involved determining the percentage fraction of positively stained cells for epithelium and tumour stroma. The cells were counted separately in central and peripheral regions of the lesions and also in the areas of normal tissues surrounding the lesions. The final data on the percentage fractions of COX-2-positive cells were presented as weighted mean values ± standard deviation. The staining intensity was also taken into consideration and was classified as weak (+; blue staining) or strong (++; dark blue or black staining).

Raw data from the present study were also analyzed using algorithms used by the authors who received contradictory results [3, 16]. Ristimaki et al. [3] scored the COX-2-positive cells and calculated percentage fractions of the cells showing moderate and/or high staining intensity. Richardsen et al. [16] assessed COX-2 expression by calculating the staining index SI defined as a weighted sum of percentage fractions of cells showing various intensity of staining calculated by multiplying the staining intensity (graded 1–3) by the percentage of positively stained cells. In the present study the staining intensity was categorized as weak or high. To ensure the comparability of our results and those of Richardsen et al. [16], the weights of 1.0 and 3.0 were assigned to the fractions of the cells showing low (+) and high (++) intensity of staining respectively.

The tumours were categorized into high versus low COX-2 expressing groups according to cut-off thresholds defined as a) a mean percentage fraction of cells expressing COX-2, regardless of the staining intensity (Algorithm 1 – ALG1), b) 10% of cells showing moderate or high staining intensity (Algorithm 2 – ALG2) as used by Ristimaki et al. [3] and c) as a median value of the SI score changing between 0–300 (Algorithm 3 – ALG3) as in the study of Richardsen et al. [16]. The three algorithms are presented in Table 2 together with resulting data concerning percentage fractions of the COX-2 expressing tumours.

**Table 2:** **Epithelial and stromal expression of COX-2 in breast cancers investigated**

	ALG 1: percentage fraction of the COX-2 positive cells (0–100) with a mean value of all the results used for recognition of tumour overexpressing the COX-2 protein						ALG 2: percentage fraction of the COX-2 positive cells (0–100) with a cut-off of 10% used for recognition of tumour overexpressing the COX-2 protein						ALG 3: percentage fraction of the COX-2 positive cells (0–100) × staining intensity (1–3) with a median value of all the results used for recognition of tumour overexpressing the COX-2 protein					
Anti-COX-2 antibody	Ab1		Ab2		Ab3		Ab1		Ab2		Ab3		Ab1		Ab2		Ab3	
Type of the cells scored (E – epithelial; S – stromal)	E	S	E	S	E	S	E	S	E	S	E	S	E	S	E	S	E	S
Cut-off value	5.1	4.2	21.1	9.8	43.0	19.0	10% of the cells with a moderate or strong staining						6.2	8.2	27.3	15.3	68.0	30.1
Percentage fraction of the tumours overexpressing COX-2	41.5	41.5	51.2	39.0	46.3	48.8	0	2.4	26.8	29.3	26.8	80.5	48.8	48.8	48.8	48.8	48.8	46.3

Staining was evaluated with three different algorithms (ALG1, ALG2 and ALG3) applied to data obtained using three different anti-COX-2 antibodies (Ab1, Ab2 and Ab3).

### Statistical analysis

The probability of overall survival was determined using the Kaplan-Meier method, and the log–rank test was used to evaluate differences in survivorship. Cox proportional hazards modeling was used to determine the value of the COX-2 expression as an independent prognostic marker. Parameters other than the COX-2 expression levels entered into Cox analyses included data on expression of HER-2 (0–3), ER (0–3), PR (0–3), lymph node status (0–1) and tumour size (1–3). All the analyses were performed with overall survival as the end point. Statistical analyses were carried out using Medcalc software package (MedCalc Software, Ostend, Belgium).

## Results

### COX-2 expression detected with three antibodies and evaluated using three different immunohistochemical scoring algorithms

**Figure 1:**
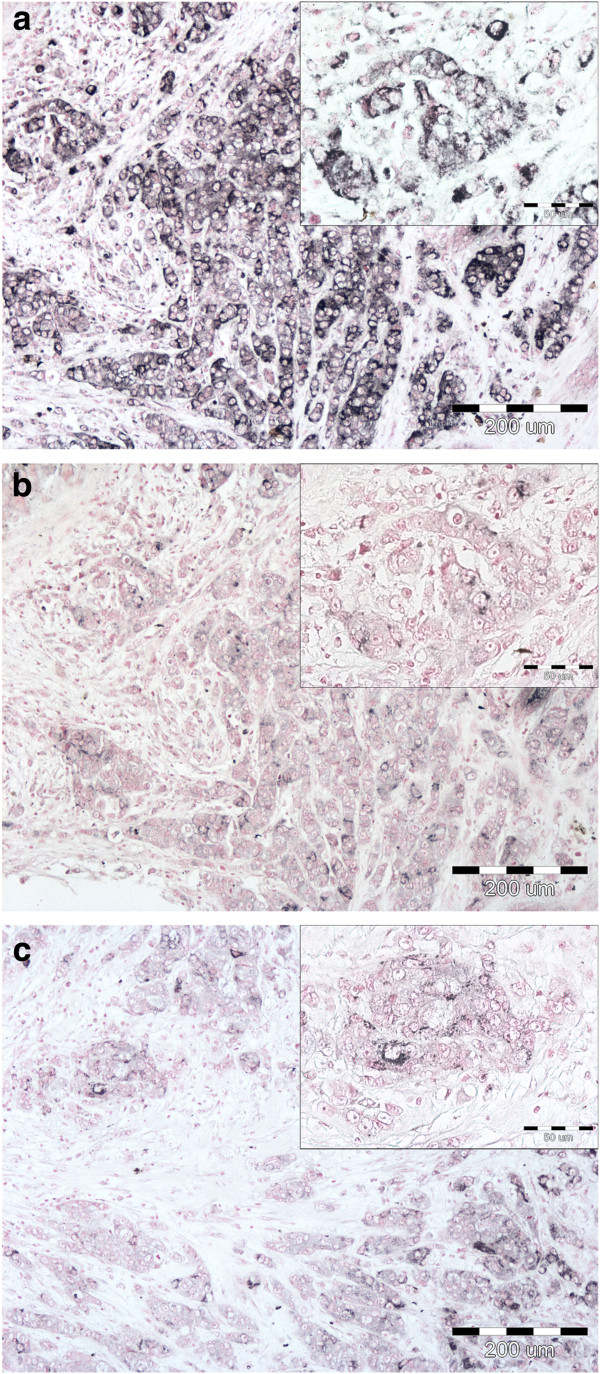
**COX-2 staining in invasive ductal breast carcinoma (T2N1).** Immunoreactivity of the polyclonal (Ab3: **a)** and monoclonal (Ab2: **b**; Ab1: **c)** antibodies, original magnifications: 100× and in insets 400 × .

All the three antibodies detected the COX-2 protein in all the lesions investigated both in the cancer cells and in the stroma (Figure 1). The detection sensitivity of the antibodies was assessed by comparing percentage fractions of the COX-2 positive cells detected in tissue sections of the same lesions. The highest detection sensitivity was observed for the polyclonal antibody Ab3 targeting the N–end fragment of the COX-2 protein. The monoclonal antibody Ab2 was ca. 50% less sensitive compared to the Ab3 antibody. Finally, the Ab1 monoclonal antibody detected COX-2 expression in very small numbers of cells usually demonstrating a low staining intensity.

For each antibody (Ab1-3) COX-2 expression levels were estimated using three different algorithms (ALG1-3). ALG1 and ALG3 classified the tumours as COX-2 positive or negative using experimentally determined cut-off thresholds (mean or median value of the score) while ALG2 based on arbitrarily determined cut-off value of 10%. Each of the three antibodies similarly differentiated between the COX-2 positive and negative tumours if either ALG1 or ALG3 were used for the evaluation of the expression of the protein within individual lesions (ALG1: 41.5%-48.8%; ALG3: 46.3%-48.8% of the COX-2 positive lesions). However, the same raw staining data interpreted using the ALG2 scoring system yielded dramatically scattered results ranging from 0% to 80% of the lesions expressing COX-2 depending on the antibody used in the experiments (see Table 2 for a brief presentation of the algorithms and the results of the classification of the tumours).

### Prognostic value of COX-2 expression in tumour epithelial cells

Kaplan-Meier analyses were carried out for the same group of tumours immunohistochemically examined for COX-2 expression using three different primary antibodies and three different algorithms selecting the tumours overexpressing the protein in the epithelial cells. The results illustrating statistical significance of a relationship between COX-2 expression and overall survival are presented in Table 3 (three upper rows).

**Table 3:** **Statistical significance of the correlations between overall survival and COX-2 expression in cancer epithelial cells**

	Ab1	Ab2	Ab3
**Kaplan-Meier analysis**			
**ALG 1**	*NS*	*0.007*	*0.032*
**ALG 2**	*-*	*0.043*	*0.020*
**ALG 3**	*NS*	*0.007*	*NS*
**Cox model including expression of ER, PR and HER**			
**ALG 1**	*NS*	*NS*	*0.017*
**ALG 2**	*-*	*NS*	*NS*
**ALG 3**	*NS*	*0.017*	*NS*
**Cox model including clinico-pathological parameters pT and pN**			
**ALG 1**	*NS*	*0.036*	*NS*
**ALG 2**	*-*	*NS*	*0.050*
**ALG 3**	*NS*	*0.001*	*NS*
**Cox model including all the parameters (ER, PR, HER-2, pT and pN)**			
**ALG 1**	*NS*	*NS*	*NS*
**ALG 2**	*-*	*NS*	*NS*
**ALG 3**	*NS*	*0.002*	*NS*

(*NS* - not significant).

**Figure 2:**
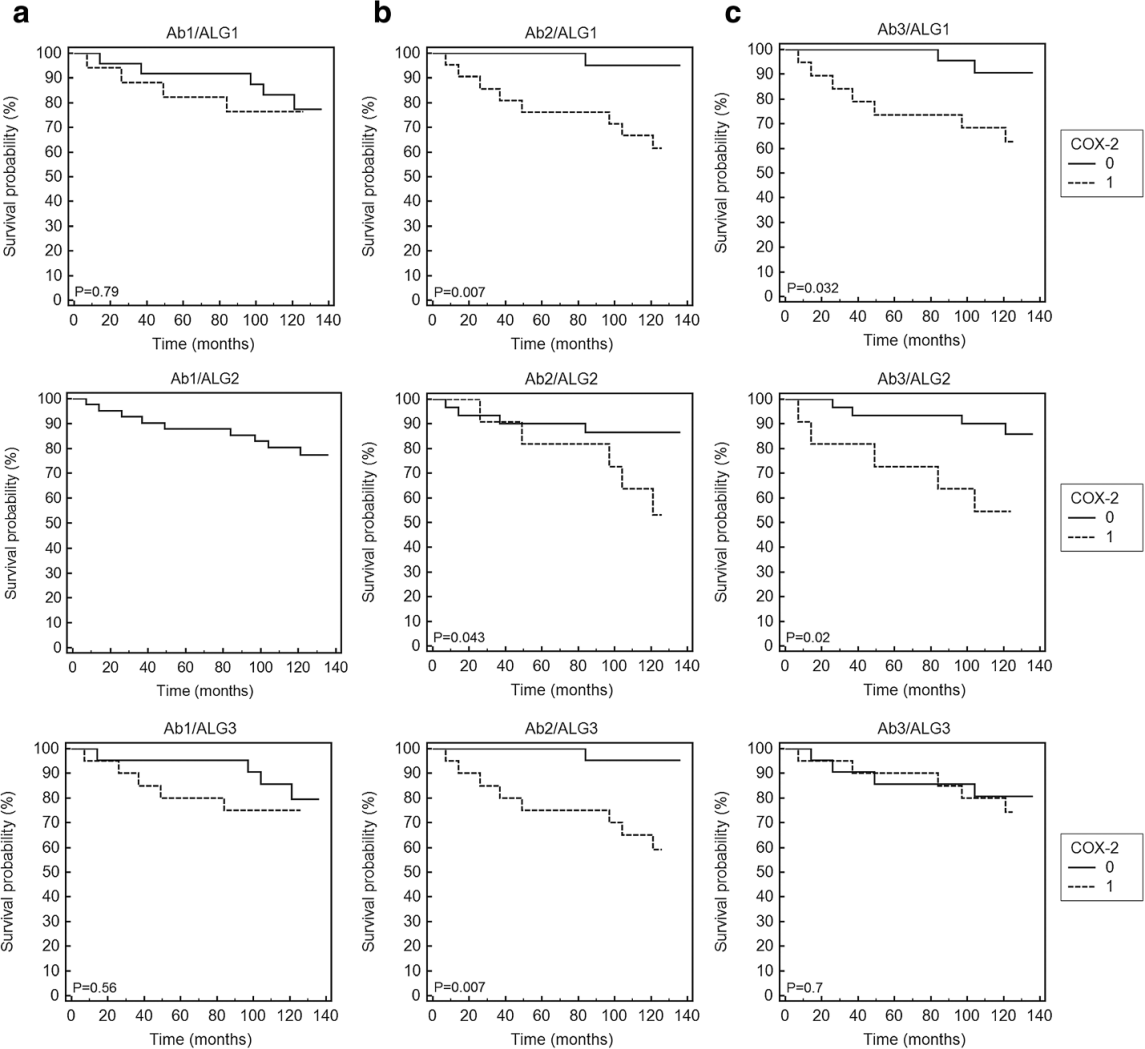
**Kaplan-Meier analysis of overall survival and COX-2 staining in the cancer epithelial cells.** COX-2 expression was detected with Ab1 **(a)**, Ab2 **(b)** and Ab3 **(c)** antibodies and evaluated using the algorithms ALG1 (upper row), ALG2 (middle row) and ALG3 (lower row). 0 – low expression; 1 – high expression.

It can be easily seen that the outcome of Kaplan-Meier analyses was strongly dependent on the type of the antibody used for the COX-2 detection. No correlation between COX-2 expression and patient survival was found using the data obtained with the Ab1 antibody, independently of the algorithm used for separating the tumours expressing and non-expressing the protein in the epithelial cells. On the other hand both the antibodies Ab2 and Ab3 yielded data demonstrating statistically significant correlation between COX-2 expression and survival with P_2_ = 0.007 and P_3_ = 0.032 respectively for the tumours selected for high COX-2 expression using ALG1 and with P_2_ = 0.043 and P_3_ = 0.02 for the same set of data analyzed with ALG2. Interestingly, using ALG3 we found statistically significant association of COX-2 expression with patient prognosis only for the data obtained with the monoclonal Ab2 antibody (P_2_ = 0.007) (Figure 2).

In all the statistically significant cases the enhanced COX-2 expression in tumour epithelial cells had negative influence on the outcome with relatively high hazard ratios (HR) for overall survival (ALG 1: HR(Ab2) = 10.01 [95% CI 2.69 – 37.29], HR(Ab3) = 4.75 [95% CI 1.26 – 17.79]; ALG 2: HR(Ab2) = 3.56 [95% CI 0.8 – 15.78], HR(Ab3) = 2.71 [95% CI 0.57 – 12.52]; ALG 3: HR(Ab2) = 10.01 [95% CI 2.69 – 37.29].

However, as shown by multivariate analysis the COX-2 expression in the cancer epithelial cells was not an independent risk factor if other variables like expression of hormone receptors ER and PR, HER-2 as well as tumour size (pT) and a presence of nodal metastases (pN) were taken into account. At such an approach analyses based on Cox proportional hazard model yielded widely varied results. The analysis including all the parameters showed statistically significant association between the enhanced COX-2 expression and worse survival only for the raw data obtained with the Ab2 antibody and processed using the ALG3 algorithm. Similar prognostic value of the COX-2 expression was also found for the raw data obtained with the Ab3 antibody processed with the ALG1 algorithm (Table 3) if only the expression of the receptors was included in the model. Models concerning only clinico-pathological parameters (pT and pN) indicated such a correlation for combinations of Ab2 with ALG1 and ALG3 or Ab3 with ALG2 (Table 3).

### Prognostic value of COX-2 expression in tumour stromal cells

**Figure 3:**
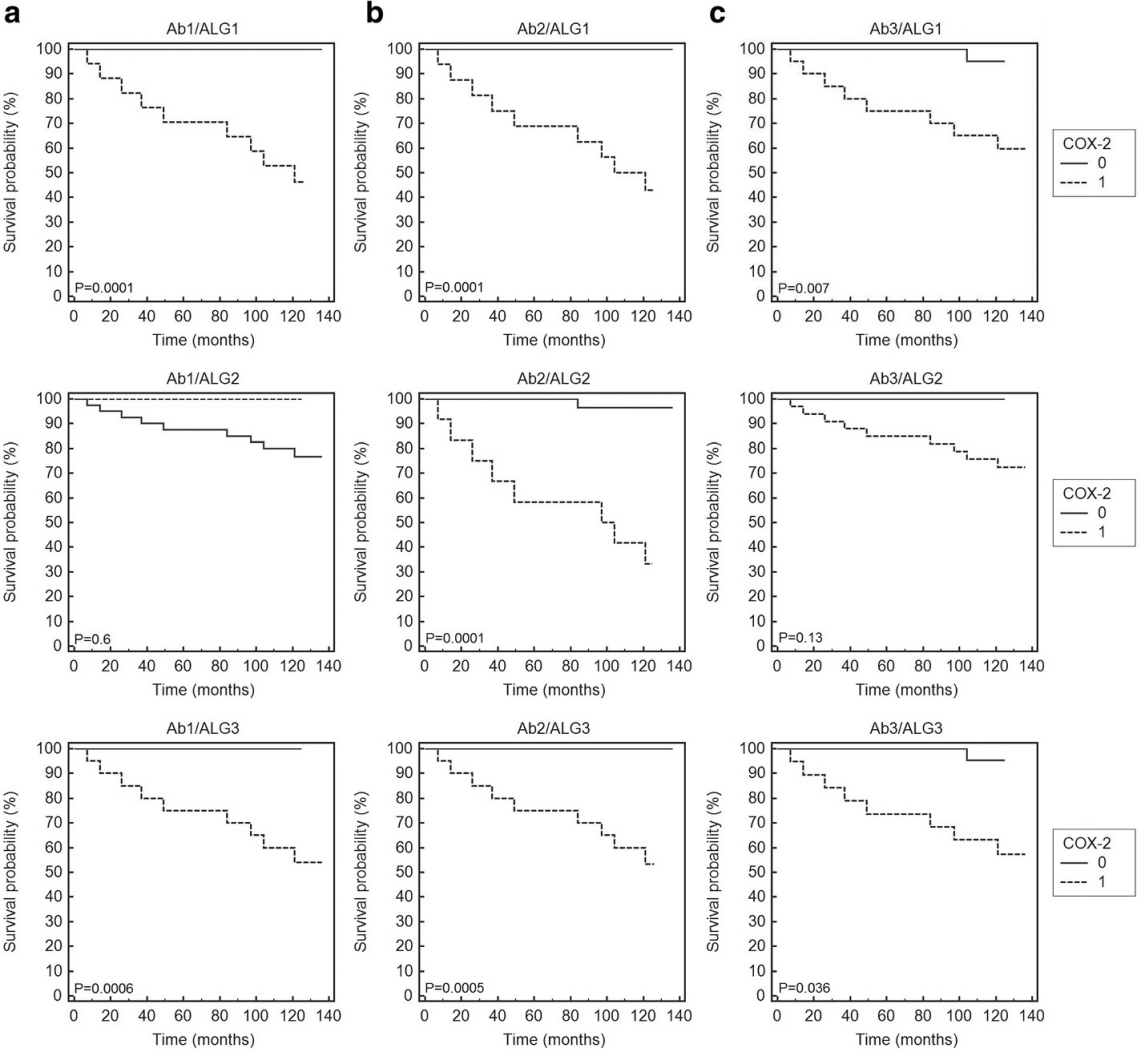
**Kaplan-Meier analysis of overall survival and COX-2 staining in the cancer stromal cells.** COX-2 expression was detected with the Ab1 **(a)**, Ab2 **(b)** and Ab3 **(c)** antibodies and evaluated using the algorithms ALG1 (upper row), ALG2 (middle row) and ALG3 (lower row). 0 – low expression; 1 – high expression.

The results obtained for the COX-2 expression in the stromal cells were more consistent compared to those found for the tumour epithelial component. Both in univariate and multivariate analyses COX-2 expression was significantly correlated with a worse survival independently of the algorithm used for processing the raw immunohistochemical data (Table 4, Figure 3). The results obtained for all the three antibodies were very similar if the raw data were evaluated using the algorithms ALG1 and ALG3. It should be noted, however, that in the case of the ALG2 algorithm the cut-off threshold defining the COX-2-positive tumours was set at a level of 10% of tumour cells demonstrating medium or strong staining intensity. According to that algorithm only one COX-2-positive lesion was detected in experiments with the Ab1 antibody while for the same group of the lesions the experiment using the Ab2 antibody showed as many as 33 (80%) COX-2-positive cases.

**Table 4:** **Statistical significance of the correlations between overall survival and COX-2 expression in cancer stromal cells**

	Ab1	Ab2	Ab3
**Kaplan-Meier analysis**			
**ALG 1**	*0.0001*	*0.0001*	*0.0070*
**ALG 2**	*NS*	*0.0001*	*NS*
**ALG 3**	*0.0006*	*0.0005*	*0.0360*
**Cox model including expression of ER, PR and HER**			
**ALG 1**	*0.0002*	*0.0001*	*0.0100*
**ALG 2**	*NS*	*0.0005*	*NS*
**ALG 3**	*0.0014*	*0.0005*	*0.0200*
**Cox model including clinico-pathological parameters pT and pN**			
**ALG 1**	*0.0006*	*0.0002*	*0.0200*
**ALG 2**	*NS*	*0.0001*	*NS*
**ALG 3**	*0.0028*	*0.0070*	*0.0360*
**Cox model including all the parameters (ER, PR, HER-2, pT and pN)**			
**ALG 1**	*0.0002*	*0.0001*	*0.0150*
**ALG 2**	*NS*	*0.0001*	*NS*
**ALG 3**	*0.0029*	*0.0040*	*0.0200*

(*NS* - not significant).

## Discussion

Variability of immunohistochemical staining with different antibodies detecting the same molecular target has been reported by several groups. Already in 2003 Garewall et al. [28] described differences in the staining patterns of three commercially available anti-COX-2 antibodies in human colonic tissues. Kuźbicki et al. [26] found significant differences in sensitivity of the three antibodies detecting the same protein in human melanoma. Similar effects were described for other widely used commercial antibodies, most recently for the antibodies used for detecting tumour-suppressor protein p16INK4a [27].

This work demonstrated considerable differences in sensitivity of immunohistochemical detection of the COX-2 protein expression in human breast cancer tissues with the three antibodies used earlier by several authors. The detection sensitivity estimated by percentage fractions of the stained cells detected in the same lesions with individual antibodies was much higher for the antibodies Ab2 and Ab3 than for the antibody Ab1.

However, the problem of the differences between the data on the COX-2 expression in breast cancer reported by different groups cannot be reduced to variability in the staining sensitivity of antibodies used. Even the groups working with the same type of primary antibodies obtained inconsistent results concerning the changes of COX-2 expression during the disease progression and the prognostic significance of the protein [3, 4, 8, 12–15, 17–19]. Therefore, in our study the raw immunohistochemical data obtained with each of the three antibodies were processed using three algorithms of assessing the levels of COX-2 expression. Two of those systems were previously used by other groups (see for instance [3, 16]).

Evaluation of a prognostic impact of the COX-2 expression revealed significant differences between the results obtained for the epithelial cancer cells and for the tumour stroma. COX-2 expression by the epithelial cells assayed with the Ab1 antibody did not provide prognostic information independently of the algorithm used for processing the immunohistochemical data. Prognostic value of the assays carried out using the Ab2 and Ab3 antibodies varied widely between the algorithms of immunohistochemical scoring. Multivariate Cox analyses entering COX-2 expression with expression of ER, PR and HER-2 and clinico-pathological characteristics of the tumours investigated yielded even more varied results. It should be noted, however, that the data obtained with Ab2 antibody and evaluated using the ALG2 algorithm yielded prognostic value of the COX-2 expression similar to that reported by Ristimaki et al. [3] who applied similar approach in the study using tissue micro-arrays.

Examination of cells of the tumour stroma demonstrated the prognostic value of COX-2 expression independently of the antibodies used in the experiments. The only exception were some of the results obtained for the raw immunohistochemical data processed with the ALG2 algorithm. A plausible cause of this discrepancy seems to be the low cut-off threshold assumed in the ALG2 for differentiating between the COX-2 positive and negative tumours. Contrary to the ALG2 the algorithms ALG1 and ALG3 do not involve arbitrary definition of the cut-off thresholds but instead “adjust” the cut-off levels to the sensitivity of the immunohistochemical reaction defining the threshold value as either a median or a mean value of the immunohistochemical score. Thus, despite significant differences in the sensitivities of the antibodies used, the ALG1 and ALG3 yielded for all the antibodies the data consistently indicating a significant correlation between enhanced levels of the COX-2 expression in the stromal cells and a worse overall patient survival. It is possible that the cut-off level of 10% of the COX-2 positive cells applied in the ALG2 algorithm according to Ristimaki et al. [3] was implicitly tuned by the authors to the sensitivity of the Ab2 antibody used in their study.

To the best of our knowledge only two groups carried out studies of the prognostic value of the COX-2 expression in the stromal cells of human breast cancer [16, 19]. Nakopoulou et al. [19] did not find significant correlation between the COX-2 expression in the stromal cells and the patient survival while Richardsen et al. [16] found that high stromal staining intensity in the primary tumours was associated with significantly higher risk of death compared to the low staining group. Our study demonstrated that stromal expression of COX-2 evaluated according to the algorithms ALG1 (this work) and ALG3 (used according to [16]) could be considered an independent prognostic factor for the breast cancer.

Interactions between cancer cells and their stroma change dynamically during disease progression and may play a role in both inhibiting and promoting tumour growth and invasiveness [24, 29]. There is a large body of data indicating that the stroma may be a major regulator of tumour growth. Also, a broad range of evidence points to a significant role of chronic inflammation in promoting tumour development although the immune system most probably also plays a role in resisting formation of neoplasias [29, 30]. The expression of inflammatory mediators leads to expression of COX-2 which in turn supports chronic inflammation by mediating tumour promoting signaling pathways [29–33]. Tumour promoting role of COX-2 has been shown in several model systems of mammary tumorigenesis [1, 34–36]. Moreover overexpression of COX-2 was found in several human cancers [1, 2]. Our immunohistochemical study with its natural limitations does not allow for determining molecular background of the discovered correlation between the enhanced stromal expression of COX-2 and worse prognosis for the breast cancer patients. It is well-known, however, that increased levels of COX-2 may be involved in controlling many cellular processes including cell proliferation and survival, inhibition of apoptosis, angiogenesis and invasiveness of the tumour cells.

## Conclusions

Our findings emphasize that the stromal, not epithelial expression of COX-2 might be an independent prognostic parameter for breast cancer. Moreover, our study demonstrates that the prognostic value of immunohistochemical assessment the stromal COX-2 expression is relatively independent of differences in sensitivities of the primary antibodies if the cut-off thresholds used for defining the cancers overexpressing COX-2 are properly tuned to the sensitivity of the antibodies used for a detection of the enzyme.

### Consent

Written informed consent was obtained from the patient for the publication of this report and any accompanying images.
